# Great apes can defer exchange: a replication with different results suggesting future oriented behavior

**DOI:** 10.3389/fpsyg.2013.00698

**Published:** 2013-10-02

**Authors:** Mathias Osvath, Tomas Persson

**Affiliations:** Department of Cognitive Science, Cognitive Zoology, Lund UniversityLund, Sweden

**Keywords:** episodic cognition, exchange, future oriented cognition, great apes, chimpanzees, orangutans

## Abstract

The topic of cognitive foresight in non-human animals has received considerable attention in the last decade. The main questions concern whether the animals can prepare for upcoming situations which are, to various degrees, contextually or sensorially detached from the situation in which the preparations are made. Studies on great apes have focused on tool-related tasks, e.g., the ability to select a tool which is functional only in the future. Dufour and Sterck (2008), however, investigated whether chimpanzees were also able to prepare for a future exchange with a human: an object exchanged for a food item. The study included extensive training on the exchangeable item, which is traditionally not compatible with methods for studying planning abilities, as associative learning cannot be precluded. Nevertheless, despite this training, the chimpanzees could not solve the deferred exchange task. Given that great apes can plan for tool use, these results are puzzling. In addition, claims that great ape foresight is highly limited has been based on this study (Suddendorf and Corballis, 2010). Here we partly replicated Dufour and Sterck's study to discern whether temporally deferred and spatially displaced exchange tasks are beyond the capabilities of great apes. In addition to chimpanzees we tested orangutans. One condition followed the one used by Dufour and Sterck, in which the exchange items, functional only in the future, are placed at a location that freely allows for selections by the subjects. In order to test the possibility that the choice set-up could explain the negative results in Dufour and Sterck's study, our second condition followed a method used in the planning study by Osvath and Osvath (2008), where the subjects make a forced one-item-choice from a tray. We found that it is within the capabilities of chimpanzees and orangutans to perform deferred exchange in both conditions.

## Introduction

The last decade has seen a number of studies on episodic-like memory and foresight in animals, primarily on corvids and great apes (e.g., Clayton and Dickinson, 1998; Raby et al., 2007; Osvath and Osvath, 2008; Martin-Ordas et al., 2010). The positive results in several of these studies suggest that the underlying cognitive system, to some extent, can be compared to the human episodic system. The human system provides the ability to remember events and cognitively construct potential future events from a subjective perspective, often including contextual elements such as “when,” “where,” and “what” information. The episodic system is usually contrasted with the semantic system, which is also declarative, but concerns general knowledge unrelated to an explicit event. (For a review on the episodic system see e.g., Szpunar, 2010).

Regardless of whether there is an episodic component associated with the future directed behaviors of the animals in question, it remains important to study such behaviors in detail. Future directed behaviors exhibited in such studies appear difficult to explain by merely associative learning of key stimuli, or by rigid mechanisms, such as fixed action patterns (e.g., Raby and Clayton, 2009; Osvath, 2010). Cognition underlying future directed behavior, which seem not purely governed by the law of effect or innate responses, epitomizes some of the hardest problems within cognitive science: how matter (i.e., the brain) can be *about* something that does not yet exist (i.e., the future). Nevertheless, we know with some certainty that many brains can do this, e.g., all those of normal adult humans.

Current methods in the research on animal cognitive foresight are influenced by views forwarded by Wolfgang Köhler in the 1920s. Köhler studied the cognition of chimpanzees, and described his observations of chimpanzees anticipating events that were “planned acts of the animal itself” (Köhler, 1921). In the cases Köhler studied, however, the rewards were always visible. That is, a key stimulus of the goal of the planning action was available for sensory feedback. Köhler argued that it would be an even bigger achievement if the chimpanzee could make preparations for events that were not yet within sight. Köhler suggested an experimental protocol for such a study: a two-room paradigm, in which one room contains a reward, and the other room holds the means of getting the reward. Access to the rooms is temporally separated.

Today, there exist only a few studies on the abilities of great apes to act toward future goals where the goals are outside the animal's current sensory scope. (Mulcahy and Call, 2006; Dufour and Sterck, 2008; Osvath and Osvath, 2008; Osvath, 2009; Osvath and Karvonen, 2012). (For studies on corvids see Correia et al., 2007; Raby et al., 2007; Cheke and Clayton, 2012, and on monkeys Bourjade et al., 2012; Dekleva et al., 2012). The experimental studies have roughly followed Köhler's protocol with the two-room paradigm. Two studies have focused on great ape abilities to select, transport and save tools that are useful only in a future context (Mulcahy and Call, 2006; Osvath and Osvath, 2008). An additional study, on chimpanzees, included the same tool-using paradigm, but also, and mainly, investigated conditions based on the ape having to select an item that after a delay of 1 h could be exchanged for a food reward from a human (Dufour and Sterck, 2008). This study relied heavily on training the animals to exchange a certain object type. It might therefore not be regarded as a planning study in a tradition in which an exclusive reliance on associative learning is precluded. This does not make the results less interesting, however.

The two studies solely based on tool use showed that chimpanzees, bonobos and orangutans are capable of selecting an appropriate tool well in advance of its use. Bonobos and orangutans could keep the tool overnight (14 h per trial) (Mulcahy and Call, 2006). One of the studies showed that orangutans and chimpanzees could disregard an immediate, but smaller, favorite reward in favor of the tool that offered the means of attaining a future, larger, reward (Osvath and Osvath, 2008). Furthermore, this study controlled for whether the apes behaved toward the tool as if it was merely a reinforced stimulus, concluding that associative learning alone could not explain the results (see also Osvath, 2010).

The study by Dufour and Sterck (2008), concentrating primarily on future exchange in chimpanzees, yielded puzzling results. In the tool-using condition, which was a replication of one part of the studies described above (i.e., Mulcahy and Call, 2006), the chimpanzees were successful. The subjects did, however, not succeed in selecting a heavily reinforced item that was usable in a future exchange with a human experimenter. Suddendorf and Corballis (2010) have forwarded that these results show that great ape foresight is surprisingly poor. Regardless of whether this is a correct assumption or not, there are at least two reasons why these results are noteworthy.

The first reason is that the results of Dufour and Sterck (2008) confirm that great apes do not merely rely on associative learning of a target item in future directed tasks. The item designated for the future exchange was reinforced a high number of times in an immediate context, i.e., in training on exchange. It was then tested and confirmed that the subjects understood its token status. Despite this extensive training in an immediate context, the chimpanzees failed to perform the exchange when a delay was introduced between the presentation of the item and the exchange event. That is, they did not “blindly” collect the objects with the most reinforcement history, which would be the prediction if associative learning of the target item alone explains the results in some of the above tests on future tool-use.

Secondly, the results might indicate that the task of deferred exchange represents a domain where future-directed cognition in chimpanzees is restricted. The authors of the study speculate that it might be a result of the cooperative nature of the task (“I give you what you want, and you give me what I want”), which is a context that in general has been suggested to be more cognitively demanding for chimpanzees than competitive contexts (e.g., Hare and Tomasello, 2004). Indeed, other studies suggest that chimpanzees can plan for agonistic encounters, and even plan for deception (Osvath, 2009; Osvath and Karvonen, 2012). The authors further consider that the exchange task might have further types of social complexity built into it that can be difficult for chimpanzees. For example, memories of the human exchange partner's reaction, e.g., as “unwilling” to give food, in cases where the chimpanzee failed to bring the correct item, might interfere with the memories of one's own choices that brought about this response. That is, it can be difficult for the animal to connect the events into a correct causal chain.

It is also important to mention that a recent study on brown capuchins (*Cebus appella*) and Tonkean macaques (*Macaca tonkeana*), using the same paradigm on future exchange, also yielded negative results (Bourjade et al., 2012). It is not clear, however, whether these results reflect the same factors that made the chimpanzees fail. Another recent study on monkeys, which to some extent was a replication of Osvath and Osvath (2008), showed that long-tailed macaques (*Macaca fascicularis*) would not select, transport and use a tool for a future purpose, not even if they received immediate cues of the reward, unless being subjected to extensive shaping (Dekleva et al., 2012). Thus, it may be that monkeys differ from great apes in their cognitive and/or learning systems underlying future-directed behavior.

Exchange tasks as such, when not deferred, usually pose few problems for great apes. The exchange of items with a human for food rewards typically develops spontaneously in chimpanzees (e.g., Hyatt and Hopkins, 1998; Brosnan and de Waal, 2005). Something that seems to require more explicit training is learning the relative values of exchangeable items (e.g., Brosnan and de Waal, 2005). Even when the reward differences are maximized (i.e., reward vs. no reward), the learning of differentially valued exchange items is not immediate (see e.g., training in Pelé et al., 2009). A further complicating factor is that an exchange situation with a social counterpart involves more than the value of items, such as judgments of the prospect of adequate reward. For example, chimpanzees typically hand out objects in an exchange only when solicited by a human, and not in the complete absence of one (Hyatt and Hopkins, 1998). That bartering is socially modulated is especially well-illustrated in studies of inequity aversion in capuchin monkeys (*Cebus apella*) (van Wolkenten et al., 2007) and chimpanzees (Brosnan et al., 2010), where a previously successfully exchanged token may be discarded, seemingly in protest, in response to the more favorable exchanges taking place between the experimenter and another subject. That the previous attempt to establish deferred exchange in chimpanzees failed might thus have its basis in social modulation, or the lack thereof, rather than the future directedness of the activities as such. A replication is therefore warranted.

In order to discern whether the ability for deferred exchange with humans is outside the cognitive scope of great apes, the current study aimed to replicate the main experiment of Dufour and Sterck (2008) with subjects with everyday experience of direct exchange with humans. We also added orangutans (*Pongo abelii*) to the pool of chimpanzees, in order to phylogenetically trace the abilities to the most distantly related great ape species to humans and chimpanzees, and to compare with the results in Osvath and Osvath (2008). Two of the subjects (one chimpanzee and one orangutan) in the current study participated also in that study. Two main conditions were used: (1) one-item-forced-selection, and (2) multiple-items-free-selection.

Condition (1), used in Experiment 1, followed the item selection procedure used in a study on planning for future tool-use by Osvath and Osvath (2008). The subject was offered to select only one of four items from a selection tray operated by a human experimenter. In the current study, one of these items had previously been reinforced in immediate exchange training. The three other objects served as distractors.

Condition (2), used in Experiment 2, was similar to the selection procedure used in Dufour and Sterck (2008), which in turn followed the procedure in Mulcahy and Call (2006). The four items were placed on the floor in a compartment, which was later opened to allow access for the subject to enter and collect any number of items. In this condition no humans were present during the selection opportunity.

This division of conditions was used because of the possibility that the different procedures might influence the results. That is, if the apes would succeed in condition (1) but not in condition (2), then the negative results in Dufour and Sterck (2008) might be explained by the method. One of the reasons for assuming a possible difference is that the performance of the apes in Osvath and Osvath (2008) seemed slightly better than in Mulcahy and Call (2006) in which multiple-items-free-selection was used. Arguably, the situation in which the animal gets one trial to choose only one item might be clearer or less distracting to the animal, than when faced with the opportunity to select several items during an extended time. The task can be said to be more structured, or “clean,” in condition (1). But despite these differences apes seem to be able to perform at a significant level when it comes to tool-using tasks in both conditions. So there likely are additional aspects of complexity when it comes to deferred exchange. One candidate factor is that the human who is present during the object choice in condition (1) represents a bartering partner, which might elicit an expectation of an exchange. Or, in a similar vein, the human constitutes a cue for the future situation where a human will also be present; so called cued recall (Berntsen et al., 2013) In condition (2), on the other hand, the presence of an object on its own, with its history of being functional in a social context, might not evoke the same actions without a triggering social cue. Alternatively or additionally, selecting in front of a human could be a form of explicit communication where the subject expresses a desire (for similar ideas on selecting for exchange in front of a human see Brosnan and de Waal, 2005). For these reasons we predicted that condition (1) would constitute a situation in which the apes had it easier to solve the task. Finally, irrespective of condition, perhaps physical contexts, like reward apparatuses, represent more concrete “futures” for an ape, than do the variable presence of humans.

## Preference testing

Before training was undertaken, subjects were tested in a selection procedure for their potential spontaneous preferences for the different items. This was done to make sure that the subject actually learned the value of the exchangeable item in the training phase. If the subject would have a spontaneous preference for the item designated for exchange, then it could superficially pass the learning criterion (see below). That is, the reason for the selection of the correct item could be the result of a spontaneous preference and not a response to training.

### Materials and methods

#### Ethics statement

All procedures were performed in compliance with relevant laws and institutional guidelines. Participation was voluntary and testing was approved by Uppsala regional ethics committee (approval no. C356/9). The Swedish Agricultural board (No. 31-2599/09) has approved Furuvik Zoo as a cognitive research facility on chimpanzees and orangutans.

#### Subjects

Two chimpanzees (*Pan troglodytes*) and two orangutans (*Pongo abelii*) participated in the study. Both chimpanzees were females, Manda and Maria-Magdalena. The orangutans consisted of one male, Naong, and one female, Dunja.

Subjects were tested at Lund University Primate Research Station in Furuvik Zoo. At the time of testing, the participants had experienced few previous experimental tests, and none requiring object exchange. One chimpanzee, Maria-Magdalena, and one orangutan, Naong, had previously taken part in a planning experiment involving selection of items from a tray (Osvath and Osvath, 2008), and the chimpanzee Manda had experience of choice procedures from participation in an object-choice task (Zlatev et al., 2013). In addition, Naong had extensive experience of various object-choice procedures, requiring the selection of items (*unpublished*). All subjects had frequent exchange experience outside of testing.

The individuals were tested in their caretaking compartments, as well as in larger indoor areas. No public visitors were present at the time of testing. No changes in feeding procedures were made and access to water was continuous. Some changes in indoor housing routines were made to minimize disturbance from group members.

#### Materials

**Figure 1:**
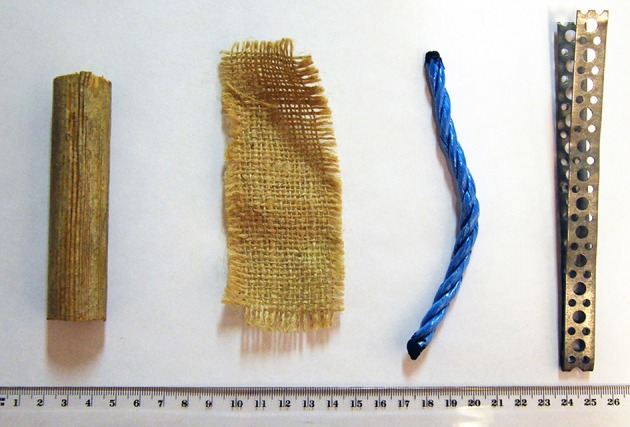
**Choice items used throughout the study**. From **left** to **right**: wooden rod, jute cloth, plastic rope, metal strip (the exchangeable item). The ruler units are in centimetres.

Four different objects were used in the selection procedures. A piece of blue plastic rope, a piece of jute cloth, a wooden rod, and a strip of bent metal (see Figure 1). The metal was the object later chosen to serve as target item in the exchange tasks (see below). The items were placed equidistantly on a 60-centimeter wide selection tray.

### Procedure

Each subject received 15 presentations of the tray baited with the four objects. The tray was slid toward the subject and was then retracted as soon as a selection had been made, thereby allowing the subject to choose only one item. A choice was scored when the animal touched or grabbed one of the objects. If the tray was retracted before the subject had managed to grasp the object, the one touched by the subject was handed over. The criterion for a “refusal” to select was when the subject entered the selection situation, looked at the items but did not attempt to touch any of them before leaving.

All subjects but the orangutan female had previous experience of this type of procedure. The order of the items was pseudo-randomized between trials. The experimenter handling the tray did not gaze at the items but slightly above or at the face of the ape.

### Results

Naong selected the piece of rope 3 times, the wooden rod 4 times, the jute cloth 1 time, and the piece of metal 3 times; in 4 trials he refused to select. The other orangutan, Dunja, selected the wood 2 times and refused to select in the rest of the trials. Manda selected the wood 12 times, the rope 2 times, and the jute 1 time. Maria-Magdalena selected the wood 12 times, the rope 2 times and refused to select once. No spontaneous preference thus existed for the metal strip at the onset of training for any of the subjects.

## Training

### Procedure

Subjects and materials were identical to the preference testing described above. The four subjects were trained until able to reliably exchange the target item (the metal strip) in a direct setting with no time delay. On the initial trials only the target item was placed in the enclosure and experimenter pointed to this and requested the “grunka” (Swedish for “the thingamajig”). Later all items were placed simultaneously into the enclosure and, pointing if needed, the experimenter asked for the “grunka.” On successful exchanges the subject was rewarded with verbal praise and a food item consisting of approximately a fifth of a banana. If the subject handed the wrong item back, the experimenter again pointed to the target item and asked for the “grunka.” In later learning trials pointing was phased out when the subject collected the target item without coaxing.

Successful learning was corroborated in tests of learning, in which 4 out of 5 trials had to be correct, which was followed by a second test (a test of retention) also requiring 4 out of 5 successful exchanges. Tests of learning and first test of retention were given on two consecutive days (except for Dunja who, due to practical reasons related to housing, received her first test of retention later the same day as she met the learning criteria).

Additional retention tests, which functioned as warm ups, were given for each new day of participation in the experimental conditions. These did not always amount to a full 5 trials before testing started, depending on how well the subject performed and/or on how motivated the animal appeared on the particular day.

In the tests of learning and retention the subject was not cued by pointing or gaze toward a particular choice object, instead the experimenter looked directly at the ape or at a point beyond it. The items were presented in a different area than the exchange. Tests of learning and tests of retention, all took place by presenting the items onto the cage floor, i.e., they were not given on a selection tray. The exception was for the female orangutan subject (Dunja) who had little previous experience of choosing from a tray, and appeared to be wary toward the tray. Thus, she received additional training trials using the selection tray. By using a test of learning and a retention test with a set criterion (80% success), we could make sure that all the subjects could perform the task in an immediate setting.

### Results

All subjects swiftly learned to hand out the target item at the expense of the other items in exchange for food (and verbal praise). The average number of trials required before we decided to test their learning against the criteria was 8.5 ± 3.9 (5–14 trials). All subjects met, and exceeded, the criteria in the subsequent test of learning: 5 out of 5 trials. All subjects also met, and exceeded, the criteria in their first test of retention: 5 out of 5 trials.

## Experiment 1—one-item-forced-selection

### Materials and methods

#### Subjects

One chimpanzee, Manda, and the two orangutans participated in this experiment. The other chimpanzee refused to take part in the experiment. The chimpanzee and one orangutan, Naong, received this experiment as their first test in the study, while the other orangutan had already been tested in Experiment 2. The chimpanzee that refused Experiment 1 had previously completed Experiment 2. Manda was tested in this experiment at the age of 5. Dunja was tested at the age of 21. Naong received his first 4 trials at the age of 20, and the other 9 trials at the age of 22. This split in time was due to circumstances unrelated to the study. We judged it to be more conservative to resume with 9 trials instead of completing a new set of 13 trials at this later occasion, as the first 4 trials could be seen as unwarranted training on the deferred task.

Naong did not receive additional training after 2 years before the 9 trials, only a test of retention, which he passed (5 out of 5). Continued good performance after a 2-year hiatus is arguably a testament to a profound ability to solve the task.

#### Materials

The selectable items in this experiment were identical to the ones used in the preference testing and training. The metal strip was the target item used in the deferred exchange for food. The food reward was approximately a fifth of a normal sized banana.

### Procedure

The procedure was similar to the one described in Preference testing. However, the experimenter (E1) operating the tray during the selection phase differed from the experimenter (E2) who performed the deferred exchange. Both experimenters were known to the subjects, but E1 had not been involved in the prior exchange training. Furthermore, the deferred exchange was made at a different location, out of sight, from the location of the selection. These additions were made to preclude sensory feedback from elements pertaining to the goal situation, other than the exchangeable item and the general presence of a human. The delay between the selection and the exchange was 15 min. The time of delay was chosen for practical reasons. The exact length of the delay plays a minor role in these types of tests as long as the selection and the exchange is parted in space and by a time span exceeding the period of storage in the working or short term memory (compare to e.g., performances of episodically amnesic patients, e.g., Tulving, 1985). After the delay, E2 showed up at the exchange location and initiated interaction with the subject. E2 held the reward, visible to the subject, in one hand and extended the other hand and asked for the item in a way identical to the training sessions. The question and gesture was repeated by E2 until it was clear that the subject had attended to the request and either acted on it or ignored it. If the subject gave the correct item to E2 he or she received the reward. The chimpanzee and the male orangutan, Naong, received 13 trials. The female orangutan, however, received 14 trials because of an unclear selection in one trial.

### Results

One chimpanzee, Manda, selected the correct object in 9 out of 13 trials (exact binomial test, *p* < 0.001). The correct selections were made in trials number 4 and 6–13. She used the correct item in the exchange 9 times (100% of the cases where the correct object was selected. Exact binomial test, *p* < 0.001). Thus, the number of complete behavioral sequences of selection, transportation and exchange was 9 out of 13 times. Given the conservative assumption that the chance for such success is 25% (due to the four selectable items), this is also significant (exact binomial test, *p* < 0.001).

The male orangutan, Naong, selected the correct object in 12 out of 13 trials (exact binomial test, *p* < 0.001). He refused to select in trial number 13. He used the correct item in the exchange 11 times (92% of the cases where the correct item was selected. Exact binomial test, *p* < 0.001). In trial number 2 he did not bring the correct item to the exchange. The number of complete behavioral sequences (11 out of 13) was significant (exact binomial test, *p* < 0.001).

The female orangutan, Dunja, selected the correct item in 11 trials out of 14 (exact binomial test, *p* < 0.001). The correct selections were made in trials number 1, 4–10, and 12–14. She refused to select in trial number 2, and refused to show up at the exchange location in 9 trials (number 1, 2, 4–6, 8, 9, 12, and 14). She never brought the correct item to the exchange location. In 4 trials (number 3, 7, 10, and 11) she tried to exchange items from the enclosure (stick, bark, and pine needles). In one of these trials (number 3) she had not selected the correct item. When Dunja had chosen the correct item, she entered into the enclosure to the male orangutan (through holes originally designed for letting gibbons, but not orangutans, through), and the male took the item from her each time (the male had already finished the two experimental conditions). See Table 1 for overview of the results.

**Table 1:** **An overview of the results in the two experiments**.

**Subject**	**Experiment 1: one-item-forced-selection**				**Experiment 2: multiple-item-free-selection**			
	**Number of selections**		**Number of exchanges**		**Number of selections**		**Number of exchanges**	
	**Correct**	**Incorrect**	**Correct**	**Incorrect**	**Correct**	**Incorrect**	**Correct**	**Incorrect**
Manda (*Pan troglodytes*)	9^***^	4	9^***^	0	12^***^	8	6^***^	0
Maria-Magdalena (*Pan troglodytes*)	Did not participate				7 n.s.	11	7^***^	0
Naong (*Pongo abelii*)	12^***^	1	11^***^	1	7^***^	1	7^***^	1
Dunja (*Pongo abelii*)	11^***^	3	0 n.s.	4	Did not enter selection room		0	2

In Experiment 1 all three subjects selected the target item significantly above chance, and two subjects exchanged the item significantly above chance (exact binomial test). In Experiment 2 two subjects selected the target item above chance, and three subjects exchanged it above chance (the latter measure is based on the coupling of the selections, including incorrect ones, with correct exchanges) (simulation in software R).

*** p < 0.001.

## Experiment 2—multiple-items-free-selection

### Materials and methods

#### Subjects

In this experiment all four subjects took part. One chimpanzee, Manda, was 7 years old, and one of the orangutans, Naong, was 22 years old, when they participated in the experiment. One of the chimpanzees, Maria-Magdalena, at the age of 11, and one of the orangutans, Dunja, at the age of 21, received this experiment as their first experiment in the study. As 2 years had transpired since Manda received Experiment 1, she was given a new set of refreshment training consisting of 5 trials including only the target item. She handed it out in all trials, as she did in the subsequent retention test.

#### Materials

The selectable items and the food reward in this experiment were identical to the ones described above. In this condition no selection tray was used, as the items were placed on the floor in a room, similar to the condition described in Dufour and Sterck (2008). However, our set-up differed slightly in that only one item per category was present, instead of the multiple items per category used by Dufour and Sterck.

### Procedure

The four selectable items were placed on the floor of a closed compartment in the everyday enclosure. A hatch was then opened and the subject was given access to enter the compartment. The hatch was left open for a 15-min selection phase with no humans present. The one handling the hatch was different from the one who later performed the exchange. The closing was followed by a 15-min delay after which the experimenter asked the subject for the exchangeable item at a different location. The exchange procedure followed the one described in Experiment 1. The behavior of the subject in the compartment baited with the items was video recorded. The video was then analyzed for the order in which the items were touched and for what items were transported out of the room.

### Results

One chimpanzee, Manda, brought the correct item out of the selection compartment in 12 out of 12 trials. Additionally she brought the rope 4 times (trial number 2, 3, 4, and 10), the wood 3 times (trial number 1, 2, 7) and the jute 1 time (trial number 1). This means that she brought the exchangeable item alone, together with no additional objects, in 5 trials. Distractor objects were never taken without also additional objects, and importantly the target object. She exchanged the correct object in 6 trials.

As the subject could select more than one item in this set-up, a standard binomial could not be used. Instead, we used Monte Carlo simulation (Robert and Casella, 2004) to calculate the *p*-value as the probability for an equal or greater number of retrievals of the correct item than that given by chance. The actual numbers of objects the subject selected were used for each of 50 000 simulated trials that were run in R (Robert and Casella, 2009). The number of selections of the exchangeable item (the metal strip) was significantly above chance (*p* < 0.001). Manda then used the correct item for exchange in 6 out of 12 trials, and did not try to exchange with any other object in the remaining 6 trials. In order to reveal a potential relationship between the selected and the exchanged item, i.e., whether she had a stronger tendency to exchange when she actually had the rewarding item, we added to the above simulation the exchange of the correct object, given the number of objects that had been selected by the chimpanzee (software R, *n* = 50.000). We considered a correct response to be an exchange of the target item, as well as refusal to trade anything if the target item had not been selected during the selection phase. This simulation provided the null distribution of the amount of correct behaviors for the subject. We then compared the observed number of correct behaviors to this distribution, and the resulting p-values represent the probability of finding an equal or greater number of correct behaviors than by chance. In other words, this gives the probability, in this particular set up, of both selecting and exchanging the correct item by chance, considering the possibility that the other selected items could be used as well. Given this method the responses of Manda were significantly above chance (*p* < 0.001). The conservative, but less representative, measure of complete behavioral sequences, from selection to exchange, is, however, not significant (exact binomial test, (*p* = 0.11). The video analysis showed that Manda, after having entered the room, touched the exchangeable item (i.e., piece of metal) first in 11 out of 12 trials.

The other chimpanzee, Maria-Magdalena, brought the correct object out of the selection room in 7 out of 12 trials (trial number 1, 3, 4, 5, 6, 7, and 9). Additionally she brought the rope 5 times (trial number 1, 8, 10, 11, and 12), the wood 4 times (trial number 1, 2, 8, and 10) and the jute 2 times (trial number 1 and 2). This means that she brought the exchangeable item alone, with no additional objects, in 6 trials, and that the rope was the only other object that was ever taken with no additional object. This was done on 2 occasions. According to the same method as described above, the number of selections of the exchangeable item was not significantly above chance (*p* = 0.094). Maria-Magdalena used, however, the correct item for exchange in 7 out of 7 possible times (100%), and did not try to exchange with any other object in the remaining 5 trials. The same method as described above revealed a significant correlation between the selected exchangeable item and the exchanged items (*p* < 0.001). The number of complete behavioral sequences, from selection to exchange, is significant (exact binomial test, *p* < 0.05). The video analysis showed that she first touched the exchangeable item in 6 trials.

One orangutan, Naong, only entered the compartment in 8 trials (trial numbers 1, 2, 3, 8, 9, 10, 11, and 12). In 7 of these trials he brought the exchangeable item. In one trial he brought the rope and the wood piece (see below). This is significantly above chance (*p* < 0.001, according to the first simulation described above). He exchanged with the correct item in 7 out of 7 possible times, i.e., when he had the metal strip available to exchange with. In one trial he tried to exchange with the wood piece. There was a significant correlation between the selected exchangeable item and the exchanged item (*p* < 0.001, according to the second simulation described above). The number of complete behavioral sequences, from selection to exchange, is significant (exact binomial test, *p* < 0.05). The video analysis revealed that he always touched the exchangeable item first, however, in one trial (number 11) he accidentally swept away the exchangeable item when he entered the room. The item was displaced outside the mesh and the orangutan tried to retrieve it without success, he then took the rope and the wood and left the compartment.

The other orangutan, Dunja, refused to enter the compartment in all trials, and refused to come to the location of exchange in all trials but 4. In 2 of these cases she tried to exchange, once with a piece of faeces and the other time with a sponge (enrichment material from the enclosure). To test whether Dunja actually understood that the compartment contained exchangeable items, three additional trials were run. In these trials she was coaxed into the room with food that was placed next to the choice items on the floor. In all cases she took the food and ignored any other items. See Table 1 for overview of the results.

## Discussion

This study did not attempt to directly study future planning, understood as an action taken in the current situation with the intention to reach a future goal, unrelated to the current psychological or perceptual state. It did, however, investigate behaviors that in effect become future oriented, and as such might constitute elements in planning acts. The goal was to discern whether great apes indeed are incapable of using an arbitrary but reinforced object to make exchanges for food with humans after a considerable delay, as has been suggested by the results in Dufour and Sterck (2008). Our results show, contrary to the previous study that it is within the abilities of both chimpanzees and orangutans. Importantly, the negative results of Dufour and Sterck can no longer be taken as evidence for “surprisingly poor” foresight capabilities in great apes, as has been done by e.g., Suddendorf and Corballis (2010).

This study, however, cannot in itself unambiguously distinguish what strategies the apes used to solve the tasks. There is a possibility that the selection of the item and the later use of it for exchange were, in a sense, cognitively separated. Given the amount of training, the selection of the item could in this case be the result of its status as a heavily reinforced stimulus. The exchange, in turn, could be a result of a combination of the reinforced action of handing out the target item to a human, and a memory for whether the item had been selected and were present in the enclosure or not. This would hold true also for the significant results that seemingly demanded coupling of correct selection with correct exchange [see e.g., (Dickinson, 2011), for a discussion on the so-called mnemonic-associative theory which can explain these types of results].

Regardless of whether one assumes merely associative learning, episodic planning, their combination, or something else, when explaining the results, they must still be contrasted to the negative ones of Dufour and Sterck (2008). The subjects of that study received a high amount of training on the exchangeable item. The value of the item must have been as associatively learned as in the current study. Thus, additional factors or mechanisms than associative learning of the value of a key item are likely needed for solving future oriented tasks like these. Something in addition to just the stimulus itself seems to be required to couple an associatively learned stimulus to a future outside the current sensory scope. This is further suggested by other studies in which the same species that were involved in the current study, indeed some of the same individuals, were shown to plan without relying on associative learning as the only or main mechanism (e.g., Osvath and Osvath, 2008; Osvath, 2010).

It is obvious that learning plays a pivotal role when it comes to couple objects with valuable exchangeability, and the situations in which such exchanges can be made. One could speculate that, apart from learning the value of the key stimuli, it might be necessary to learn more abstract relationships. For example that humans are reliable exchange partners over time. To be able to perform deferred exchange the ape might need more experiences, in several contexts, of humans as stable bartering partners. Acting future orientedly toward static objects in the environment, e.g., a nut tree or a reward apparatus, even if they are out of sight, could be a simpler task as such things are less changing in comparison to the dynamic behaviors of others.

No systematic differences between the results of Experiment 1 and 2 could be found. As the conditions differ in setup and require different statistics, direct comparisons are not possible. A comparison would only be useful if, across subjects, one condition would yield significant results and the other would not. Even then, given the few number of subjects of this study, it would still only be an indication. Nevertheless, it was shown that apes could successfully manage both conditions, and, despite the small number of subjects, the study thus answers the question of whether deferred exchange is possible for great apes in the affirmative.

Two subjects in this study, Maria-Magdalena and Naong, also participated in a study on planning for tool use (Osvath and Osvath, 2008). In the current study Maria-Magdalena participated only in one condition, which is not immediately comparable to the selection phase in the baseline condition in Osvath and Osvath. However, one can use a conservative measure, by calculating the number of successful complete behavioral sequences, from selection to use of the items. When comparing the results of the baseline condition in Osvath and Osvath (2008)—where a functional tool had to be selected and transported to a different location in the future—with the results in the current study, both subjects performed significantly above chance in both studies (exact binomial tests, *Maria-Magdalena* Experiment 2, this study: *p* < 0.05; in the baseline in the previous study: *p* < 0.001; *Naong* Experiment 1, this study: *p* < 0.001; Experiment 2: *p* < 0.05; baseline in the previous study: *p* < 0.001).

When it comes to the poor performance of the female orangutan, Dunja, one can only speculate. There could be several reasons for her results. She did indeed select the correct item in Experiment 1 significantly above chance, but did not get the chance to show whether she would exchange it or not in the future, as she after selection always returned to the male who took the item. One could of course reason that such behavior would be a sign of poor maximation of future rewards, but it could also be that the company of the male was more rewarding than a fifth of a banana. Interestingly, she did not ever attempt to take the item in Experiment 2, not even when coaxed into the room containing the objects. It can be that the mere presence of a human—a close up and potentially interactive partner—was the relevant factor for success, but we cannot know. There is one conspicuous difference in background between Dunja and the rest of the subjects. Dunja arrived at the zoo only 1 year before the study, while the rest of the subjects had been at the zoo for many years. At Furuvik Zoo the apes routinely exchange objects for food with the caretakers. This is a skill the zoo upholds in order to make the apes pick up objects from inside the compound that should not be there. When it comes to Dunja the background is less clear. Her previous caretakers reported that she did spontaneously and frequently engage in exchanging objects (mainly faeces) for food rewards at her former zoo from a young age. Without targeted studies, however, we do not know if there are systematic differences in the exchange behaviors between the two zoo environments. There might be a possibility that a difference in long-term experiences could explain the results. Relating to this issue, it would be interesting to know the background experiences of the chimpanzees in the study of Dufour and Sterck (2008).

In any case, this study shows that it is within the capacity of apes to succeed in deferred exchange. More studies are needed to understand more precisely what type of future oriented tasks apes can solve and why, including the possible effect of individual background experience.

### Conflict of interest statement

The authors declare that the research was conducted in the absence of any commercial or financial relationships that could be construed as a potential conflict of interest.
